# Deoxynivalenol Detection beyond the Limit in Wheat Flour Based on the Fluorescence Hyperspectral Imaging Technique

**DOI:** 10.3390/foods13060897

**Published:** 2024-03-15

**Authors:** Chengzhi Wang, Xiaping Fu, Ying Zhou, Feng Fu

**Affiliations:** 1 School of Information Science and Engineering, Zhejiang Sci-Tech University, Hangzhou 310018, China; 202120602088@mails.zstu.edu.cn; 2 Key Laboratory of Transplanting Equipment and Technology of Zhejiang Province, Hangzhou 310018, China; 3 Hangzhou Customs Technology Center, 398 Jianshe San Road, Hangzhou 311202, China; zhouy@zaiq.org.cn; 4 School of Computer Science and Technology, Zhejiang Sci-Tech University, Hangzhou 310018, China; fufeng@zstu.edu.cn

**Keywords:** fluorescence hyperspectral imaging, wheat flour, DON infection, machine learning, deep learning

## Abstract

Deoxynivalenol (DON) is a harmful fungal toxin, and its contamination in wheat flour poses a food safety concern globally. This study proposes the combination of fluorescence hyperspectral imaging (FHSI) and qualitative discrimination methods for the detection of excessive DON content in wheat flour. Wheat flour samples were prepared with varying DON concentrations through the addition of trace amounts of DON using the wet mixing method for fluorescence hyperspectral image collection. SG smoothing and normalization algorithms were applied for original spectra preprocessing. Feature band selection was carried out by applying the successive projection algorithm (SPA), uninformative variable elimination (UVE), competitive adaptive reweighted sampling (CARS), and the random frog algorithm on the fluorescence spectrum. Random forest (RF) and support vector machine (SVM) classification models were utilized to identify wheat flour samples with DON concentrations higher than 1 mg/kg. The results indicate that the SG–CARS–RF and SG–CARS–SVM models showed better performance than other models, achieving the highest recall rate of 98.95% and the highest accuracy of 97.78%, respectively. Additionally, the ROC curves demonstrated higher robustness on the RF algorithm. Deep learning algorithms were also applied to identify the samples that exceeded safety standards, and the convolutional neural network (CNN) model achieved a recognition accuracy rate of 97.78% for the test set. In conclusion, this study demonstrates the feasibility and potential of the FHSI technique in detecting DON infection in wheat flour.

## 1. Introduction

Wheat is one of the principal cereal crops for human consumption and has the highest cultivation coverage and production volume worldwide [1]. It can be processed into diverse foodstuffs following milling or beverages. Nonetheless, wheat plants are susceptible to infection by *Fusarium fungi* during the growth period, which induces Fusarium head blight (FHB). FHB not only causes significant yield losses but also results in the production of fungal toxins, including deoxynivalenol (DON), by pathogenic fungi. Consequently, wheat grains and their processed products are easily contaminated by such fungal toxins [2,3]. DON can cause vomiting, diarrhea, and immunosuppression in both humans and animals upon consumption of grains and their processed products containing this fungal toxin. Due to its strong immune toxicity and thermal stability, the presence of DON residue in wheat products causes a serious concern for food safety. Many countries and international organizations around the world, like the Codex Committee on Contaminants in Food (CCCF) of the United Nations (UN) Codex Alimentarius Commission, have established strict regulations or set up standards on the permissible concentration of DON in food [4].

Multiple techniques, including enzyme-linked immunosorbent assay (ELISA) [5] and high-performance liquid chromatography (HPLC) [6], have been successfully employed for DON concentration detection. Notably, HPLC is the standard detection method. Despite the accuracy of these chemical analysis procedures in identifying trace amounts of DON in samples, they are complicated, time-consuming, and destructive, and they require expensive instruments [7]. In research on low-cost detection methods, sensors are one of the hot topics, such as screen-printed electrodes that reduce impedance by specifically recognizing DON molecules and competitive immunosensors that identify DON through bioactive molecules [8,9]. These sensors may have low costs, high specificity, and high precision in detecting vomitoxin in wheat flour, but they still require the use of chemical reagents for the destructive extraction treatment of samples, which is not conducive to high-throughput testing. Consequently, a straightforward and high-throughput technique for the detection of DON in grains needs to be developed.

The spatial and spectral information of a measured object can be simultaneously obtained through hyperspectral imaging (HSI). By analyzing abundant high-dimensional spectral information, researchers can obtain the relative physical and chemical properties of the measured object. The technique’s outstanding ability for nondestructive detection in various fields has been extensively reported [10,11]. Despite severe FHB infection in the wheat crop, the DON content in wheat grains remains low, at only a few milligrams per kilogram (mg/kg) [12]. Nevertheless, near-infrared spectroscopy is a potential method for detecting the DON content. Wheat grains infected by Fusarium fungi exhibit external changes such as color whitening and grain shrinkage. Furthermore, wheat grains undergo varying changes in protein, fat content, and other chemical properties. These changes indirectly indicate the presence of DON residue in wheat grains, providing a theoretical basis for the quantitative or qualitative detection of DON residue via near-infrared spectroscopy [13]. A recent study [14] indicated that HSI is feasible and effective for the quantitative analysis of the DON content in individual wheat grains. The root mean square error of prediction (RMSEP) of a local partial least-square (PLS) model based on global PLS scores (LPLS-S) was 40.25 mg/kg, which is a favorable result for wheat grains that have the highest DON content of 507 mg/kg. In another study, Liang et al., (2020) [15] established models based on HSI data to classify wheat grains and wheat flour with DON residue, using 1 mg/kg as the classification threshold. The accuracy of the test sets were 100% and 96.4%, respectively. The above-mentioned research indicates the potential of the HSI technique to differentiate between infected and normal wheat. However, the presence of DON in normal wheat grains can occur due to the transfer of the DON substance in wheat grains. Moreover, other factors such as diseases, environmental variations, and processing methods during the natural cultivation and postprocessing of wheat can cause changes that resemble fungal toxin infections in wheat grains and flour. Consequently, considering the alterations in wheat grain or wheat flour composition as a basis for DON presence appears to be imperfect [16,17]. As such, finding a high-throughput method that can directly detect the DON content in grains is of great significance.

Fluorescence hyperspectral imaging (FHSI) is a type of technique capable of capturing fluorescence spectra and spatial information of a target object [18]. When fluorescent materials are illuminated with a specific wavelength of light, their atoms or molecules absorb energy at the excitation wavelength, which causes the electrons to undergo a transition from a low-energy state to an excited state and then back to a low-energy state, thereby emitting radiation with a longer wavelength [19]. The fluorescence intensity of the spectral image, emitted by fluorescent substances, varies with their chemical composition and concentration level. As a result, fluorescence spectra are commonly utilized for both the qualitative and quantitative detection of certain substances. Fluorescence imaging is considered a more sensitive optical sensing method than reflection imaging, which is primarily due to the greater dynamic response of fluorescence imaging to changes in compound concentration, especially subtle changes [20]. Over the last twenty years, the FHSI technique has been applied in many detecting aspects, like microbial detection [21], biological product quality assessment [22], plant chlorophyll of agro-products [23], and so on.

Many fungal toxins exhibit fluorescence, such as aflatoxins, which emit a bright yellow-green fluorescence under ultraviolet light. Samples contaminated with aflatoxins exhibited characteristic peaks on their fluorescence spectra, completely different from normal samples, while no such differences appeared on their diffuse reflectance spectra [24]. This indicates that mycotoxins with strong fluorescence effects can be identified directly by analyzing the distinctive characteristic peaks in their fluorescence spectra. In another study [25], the researchers used either naturally infected or artificially contaminated figs with aflatoxins. The results indicated that the FHSI technique identified naturally contaminated samples accurately. However, the toxin added artificially was undetectable because it was absorbed onto the dry surface of the fruit, which prevented it from emitting the characteristic fluorescence of the mycotoxin. In another study, the researchers applied a solution of aflatoxin onto the surface of peanuts and gathered FHSI data. Based on the analysis of fluorescent signals, the pixels representing toxins on the surface of peanuts were effectively identified [26]. On the other hand, infected cereal plants and their seeds usually manifest visible changes in appearance. However, in cereal products such as wheat flour, this ability may be lost or weakened after grinding, thereby increasing the challenge of detecting mycotoxins. Studies using the FHSI technique have been reported for the detection of powdered materials, including the effective classification of flour with similar properties [27,28]. From the perspective of food safety evaluation, the detection of the mycotoxin content in wheat flour using FHSI is more meaningful. Currently, there is no relevant research conducted using the FHSI technique to detect the DON concentration in wheat flour.

The aim of this study was to explore the feasibility of using the FHSI technique for the detection of excessive DON content in wheat flour. Specific objectives were to (1) analyze the fluorescence characteristics of wheat flour samples prepared artificially with varying concentrations of DON; (2) discriminate the wheat flour samples with excessive DON infection based on fluorescence spectra; and (3) find a better method for detecting excessive DON in wheat flour by comparing different methods of spectral preprocessing, feature band selection, and qualitative modeling.

## 2. Materials and Methods

### 2.1. Sample Preparation

The DON substance was purchased from Aladdin Biochemical Technology Co., Ltd. (Shanghai, China), and fluorescence spectral analysis was conducted using an FLS980 fluorescence spectrophotometer (Edinburgh Instruments, Kirkton Campus, Livingston, UK) at a professional testing company (Zheda Femtosecond Detection Technology Co., Ltd., Hangzhou, China). DON aqueous solutions were prepared in concentrations of 5, 25, and 100 μg/mL for fluorescence spectral analysis.

Wheat flour (“Xinliang” brand, product standard number GB/T 1355) was purchased from a local supermarket, with a quality grade of “special one”. The DON aqueous solutions were prepared at varying concentrations and then mixed with the wheat flour to prepare wheat flour samples with different DON concentration levels (0~1.0 mg/kg with the gradient of 0.1 mg/kg; 1.1~1.7 mg/kg with the gradient of 0.2 mg/kg; 2.0~4.0 mg/kg with the gradient of 0.5 mg/kg; 5.0 mg/kg; and 6.0~10 mg/kg with the gradient of 2 mg/kg; in total, 72 individual samples, with three replicates for each concentration). The mixed wheat flour samples were then dried, milled, and lightly filled (not compacted) into quartz dishes with an inner size of 40 mm × 40 mm × 4 mm. The excess wheat flour on the surface was gently scraped off using a ruler.

According to the national standard GB2761-2011 [29], the limit for DON in cereals and cereal products is 1 mg/kg. Hence, this study considered 1 mg/kg as the threshold to determine if wheat flour contains excessive toxins. Wheat flour samples with DON concentration greater than or equal to 1 mg/kg were considered positive samples, and those with less than 1 mg/kg DON were negative samples.

### 2.2. Hyperspectral System and Data Acquisition

**Figure 1:**
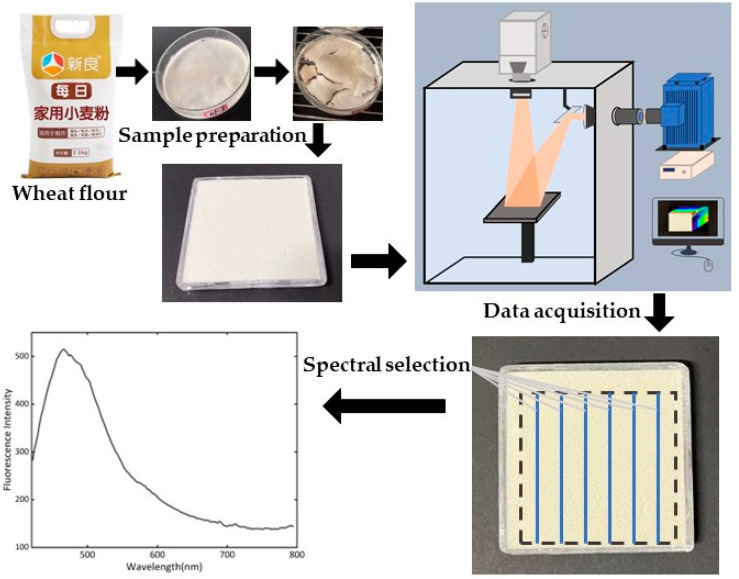
Schematic of the FHSI system and data acquisition process.

The core part of the FHSI system was a hyperspectral camera (GaiaField-V10E-AZ4, Sichuan Dualix Spectral Image Technology Co. Ltd., Chengdu, China), with a spectral range of 380–1000 nm and a spectral resolution of 2.8 nm. The hyperspectral camera included a 16-bit scientific complementary metal–oxide–semiconductor (SCMOS) detection elements with 2048 × 2048 pixels (Zola 4.2, ANDOR, London, UK), an imaging spectrometer (V10E, SPECIM, Oulu, Finland), and a C-mount lens (23 mm focal length). Another main part of the FHSI system was a 500 W xenon lamp light source (GLORIAX500A, Sichuan Dualix Spectral Image Technology Co. Ltd., Chengdu, China), with a light spectral range of 250 nm–2500 nm. Band-pass and high-pass filters were placed, respectively, in front of the light source and camera lens. The FHSI system also had an adjustable sample stage with a size of 300 mm × 300 mm. Image acquisition was conducted in a black box, using the hyperspectral image acquisition and data analysis software developed specially for the FHSI system (SpecSight, Sichuan Dualix Spectral Image Technology Co. Ltd., Chengdu, China). Figure 1 shows the schematic diagram of the FHSI system used in this study.

After the xenon lamp was properly preheated (about 15 min), the quartz dish containing the wheat flour was placed in the center of the excitation light irradiation area. This position was marked to ensure that each sample was placed at this position for image acquisition to reduce the influence of uneven excitation light. One sample was detected at each time. In order to capture a stronger fluorescence signal, the camera exposure time was set to 800 ms. Fluorescence hyperspectral image data processing and analysis were performed using MATLAB (Version: 9.0.0.341360, R2016a, MathWorks, Natick, MA, USA) and Python 3.8.

### 2.3. Hyperspectral Data Preprocessing

Due to the phenomenon of second excitation in the fluorescence spectra [30] of wheat flour samples after 800 nm, hyperspectral data in the range of 420–795 nm were extracted for further analysis. The method for extracting the region of interest (ROI) was as follows: (1) A fixed coordinate system was used to extract the effective area of the sample. (2) Within this rectangular area, one column of pixels was selected from every ninth column of pixels, and thus nine columns of pixels in total were selected for calculating the average spectra. (3) Ten such average spectra were extracted for each sample to reduce the impact of uneven excitation light intensity in the illumination area. The diagram in Figure 1 shows the extracted spectra. In total, 720 spectral curves were extracted for modeling analysis.

The image data collected using the FHSI system sometimes may be affected by system and environmental noise. In this study, several preprocessing algorithms such as normalization, multiplicative scatter correction (MSC), and Savizkg–Golag smoothing (SG smoothing) were chosen to reduce spectral noise and improve the signal-to-noise ratio of the fluorescence signals. Additionally, the optimal algorithm was selected based on the accuracy of the established models [31].

### 2.4. Hyperspectral Data Analysis

#### 2.4.1. Optimal Band Selection

The hyperspectral image data in the region of 420–795 nm include 149 wavelengths, which still have high-dimensional and redundancy information. Many studies have proved that selecting bands from high-dimensional spectra data can effectively improve model performance and reduce modeling time [32]. In this study, four band selection algorithms, namely the successive projection algorithm (SPA), uninformative variable elimination (UVE), competitive adaptive reweighted sampling (CARS), and the random frog algorithm, were used for feature waveband selection.

The SPA is a forward iterative search algorithm that selects a band with the longest projection vector by adding wavelength variables in each iteration and chooses a set of feature bands with the lowest redundancy based on the calibration model [33]. UVE adds a white noise vector with the same dimension as the independent variable to the model and deletes the variables with low contributions based on a comparison between the covariances of each variable. The remaining variables’ combination is the desired feature bands [34]. CARS first selects a certain proportion of the dataset according to the Monte Carlo model and obtains the absolute regression coefficients of the variables. Then, it uses a band selection method based on the exponential decay function and adaptive reweighted sampling to remove variables iteratively. Multiple band subsets are obtained through iterative cycles, and the final set of feature bands is determined based on the results of cross-validation [35]. The random frog algorithm is based on a reversible jump Markov chain Monte Carlo method and optimized through iteration. It randomly selects an initial variable subset and accepts it with a certain probability; finally, the selection probability of each variable is computed as a measure of the importance of feature bands [36].

#### 2.4.2. Modeling Methods

Random forest (RF), support vector machine (SVM), and CNN methods were used for modeling.

RF is an ensemble learning algorithm based on decision trees. It predicts the label values of samples by aggregating a set of n (n ≥ 1) randomly grown trees. In classification problems, the average relative frequency of class labels in the terminal nodes of the RF is used as the predicted probability for each class label. The randomness of the RF is reflected in two points: (1) Each decision tree uses a randomly extracted sample set from the training set; (2) during the process of growing decision trees, each node randomly selects a number of feature bands that is less than the total number of feature bands and uses the feature variable with the best segmentation ability to split the node [37]. Some studies have reported the use of RF models to solve classification problems for fluorescence hyperspectral data, such as categorizing oolong tea varieties [38] and distinguishing peanut surfaces contaminated with aflatoxin [39]. In this study, the number of trees was set to 500.

The SVM algorithm is one of the best solutions for binary classification problems. It maps the feature vectors to some points in space and looks for a line or a surface to differentiate these two classes of points in that space [40]. In this research, the radial basis function (RBF) was chosen as the kernel function for the SVM, a fixed penalty coefficient c was used, and the parameter g was called within the range of 10^−6^ to 10.

**Figure 2:**
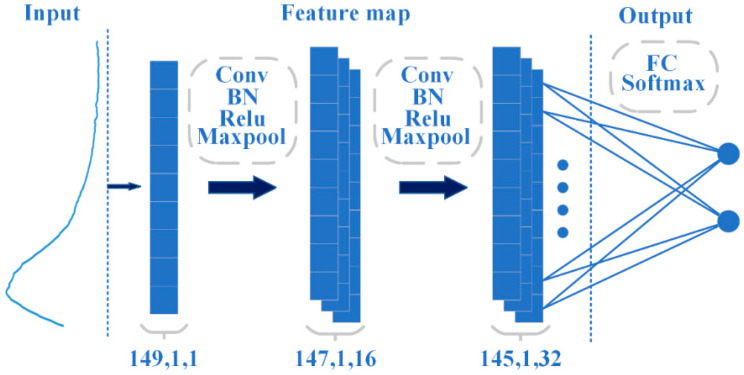
Diagram of CNN network structure.

The CNN algorithm is one of the core algorithms of deep learning, which consists of multiple convolutional layers, pooling layers, and a fully connected layer to extract features and classify data within the “receptive field” [41]. The CNN structure used in this study is shown in Figure 2. After feature extraction using a convolutional layer (with a kernel size of 2 × 1), spectral data underwent batch normalization to normalize the features and improve network generalization while accelerating convergence. Then, the ReLU activation layer and maximum pooling layer (with a stride of 1 and step of 2) were used to reduce parameters and prevent model overfitting [42].

#### 2.4.3. Model Performance Evaluation and Spectral Feature Visualization

The Kennard stone (KS) algorithm was used to divide the training and test sets in a 3:1 ratio [43]. The discrimination accuracy (Accuracy) of the test sets, as well as the recall rate and false-positive rate (FPR) of the test set, were used as quantitative indicators for model performance evaluation. The formulas for calculating these indicators are as follows:

(1) $$\mathrm{Recall}=\frac{TP}{\left(TP+FP\right)}$$

(2) $$\mathrm{FPR}=\frac{FN}{\left(TN+FN\right)}$$

where TP, FP, FN, and TN represent true-positive, false-positive, false-negative, and true-negative samples.

The t-distributed stochastic neighbor embedding (t-SNE) algorithm is an effective method for visualizing high-dimensional datasets that require classification [44]. This algorithm converts the similarity between data points into conditional probabilities and represents the data points as a Gaussian joint distribution in the original space, which is then transferred to a t-distribution in the embedded space. The algorithm uses gradient descent to minimize the loss function to obtain the converged result. In this study, t-SNE was used to visualize both the original spectral data and the features extracted by the CNN.

## 3. Results

### 3.1. Determination of DON’s Excitation and Emission Wavelengths

**Figure 3:**
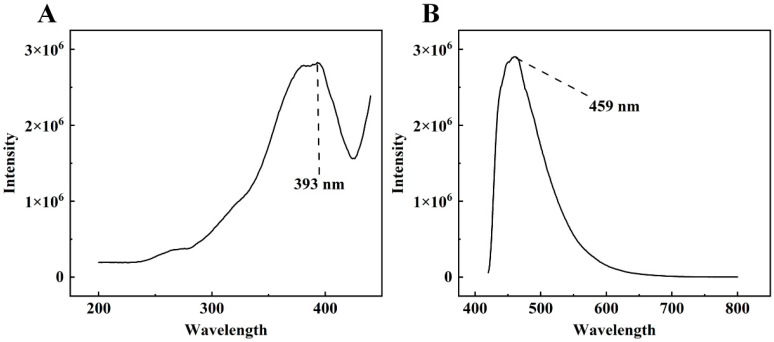
The excitation spectrum (**A**) and emission spectrum of the 100 μg/mL DON aqueous solution (**B**).

The excitation and emission wavelengths of DON were tested using an Edinburgh FLS980 fluorescence spectrometer. Figure 3 shows the excitation spectrum and the emission spectrum of a 100 μg/mL aqueous solution of DON. As the figure shows, DON emits fluorescence when excited by light at the appropriate wavelength. The maximum fluorescence intensity was obtained at about 460 nm (exactly 459 nm in this study) when the excitation wavelength was chosen near 390 nm (exactly 393 nm in this study). Therefore, the excitation and emission wavelengths of DON were determined to be 393 nm and 459 nm, respectively.

During the acquisition of the fluorescence hyperspectral images of wheat flour, a band-pass filter (half-band width = 30 nm) with a central wavelength of 390 nm was placed in front of the light source, and a high-pass filter with a cut-off wavelength of 420 nm was placed in front of the camera lens.

### 3.2. Fluorescence Spectra of Wheat Flour Containing Different Concentrations of DON

**Figure 4:**
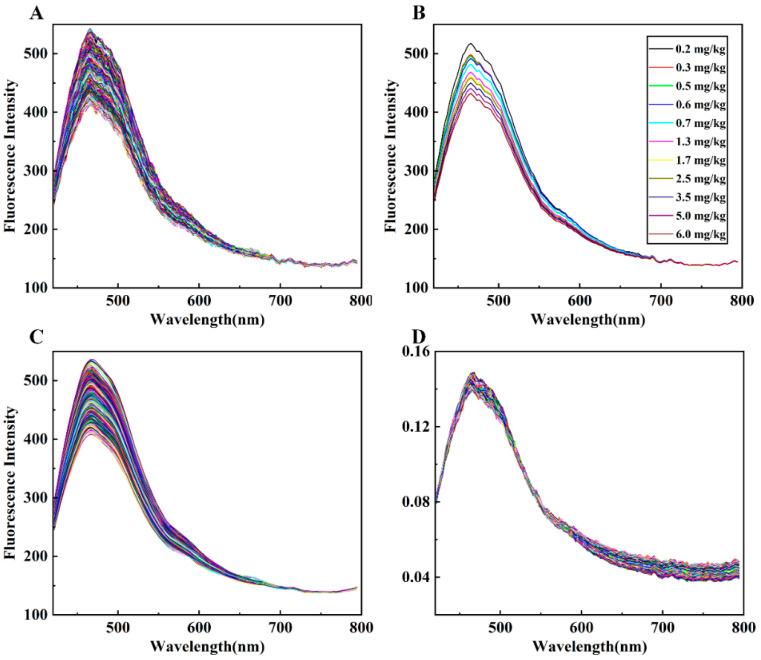
The average spectral curves of wheat flour containing different concentrations of DON: (**A**) original spectra; (**B**) spectra corresponding to 11 concentrations of DON that meet the concentration and intensity change regularity of DON; (**C**) spectra processed by SG smoothing; (**D**) spectra processed by normalization.

After collecting the fluorescence hyperspectral images of all wheat flour samples and calculating the average spectra, 720 original sample curves were obtained, as shown in Figure 4A. The original spectral intensity was observed to be notably low with substantial noise, due to the weak fluorescence effect of wheat flour. The spectral trends of fluorescence among all samples were alike, with each presenting a peak at 468 nm and a smaller secondary peak at 478 nm. Wheat flour primarily consists of approximately 75% starch (70% amylopectin and 30% amylose); 15% protein (such as glutenin and gliadin); and some trace components, including various vitamins. The majority of these components exhibit fluorescence properties. For instance, researchers found that glutenin and gliadin have excitation/emission wavelengths of fluorescence around 290 nm/350 nm [45,46]. The wheat flour fluorescence characteristic observed in this research might be caused by the excitation of amylopectin. Additionally, the accompanying trace components such as vitamin B1 and riboflavin share comparable fluorescence properties.

From a visual perspective, the spectral differences in wheat flour samples with varying concentrations of DON were mainly reflected in intensity differences. In general, wheat flour samples with higher concentrations of DON exhibited lower peak fluorescence intensity. The average spectral peak intensities of the DON samples, measured at each concentration, were observed and compared, as illustrated in Figure 4B. The average spectral peak intensity of the samples decreased with increasing concentrations of 0.2 mg/kg, 0.3 mg/kg, 0.5 mg/kg, 0.6 mg/kg, 0.7 mg/kg, 1.3 mg/kg, 1.7 mg/kg, 2.5 mg/kg, 3.5 mg/kg, 5.0 mg/kg, and 6.0 mg/kg. However, the spectra of other concentrations did not strictly follow this rule, with confusion at similar concentrations. The spectral curves after SG smoothing and normalization preprocessing are depicted in Figure 4C,D, respectively. It can be seen that the spectral features of the normalized samples after 600 nm are amplified, and some features at 470 nm are preserved.

Modeling analysis using SVM and RF methods was conducted on the original spectra and spectra processed with five distinct preprocessing methods. The results, as shown in Table 1, indicate that the different preprocessing methods induce unique impacts on the performance of the wheat flour classification models. The MSC preprocessing method significantly improved the recall of the model, especially in the SVM model, with a recall rate of up to 96.84%. Nevertheless, the FPRs were in both models. Upon thorough comparison, the data processed through the normalization and SG smoothing preprocessing methods exhibited improvements in the model’s performance. Thus, these two methods were selected for data preprocessing in further analysis.

### 3.3. Feature Band Selection Results

**Figure 5:**
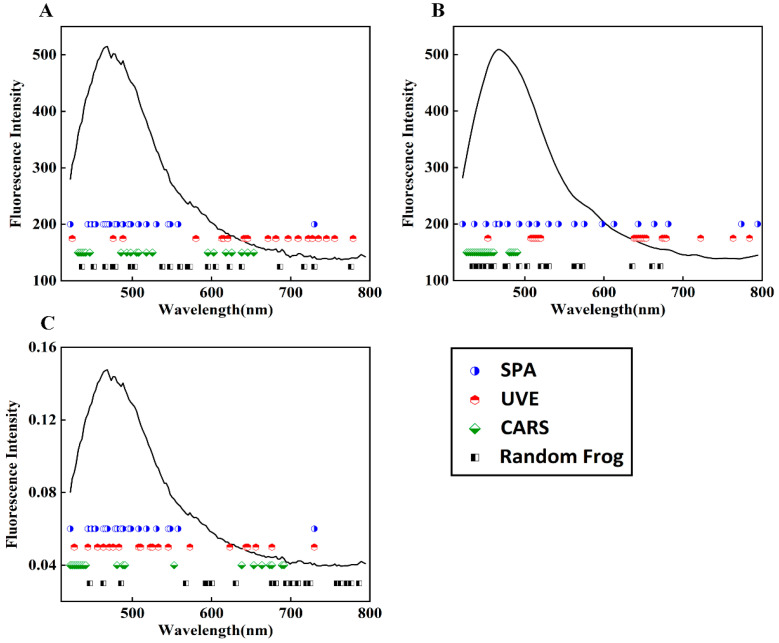
Four algorithms selected bands to dimensionally reduce spectral data that underwent three preprocessing methods: (**A**) no preprocessing; (**B**) SG smoothing preprocessing; (**C**) normalization preprocessing.

When using the SPA, UVE, CARS, and random frog algorithms to select the feature bands from the fluorescence spectral data, the main parameters of each algorithm were determined based on preselection and comparison results. The Monte Carlo simulation was set to 500 for UVE, and the modeling set proportion was set to 0.7. The Monte Carlo sampling times were set to 100 for CARS, and the verification method was five-fold cross-validation. The iteration times for the random frog algorithm were set to 1000. For ease of comparison between model performances, the 20 bands with the highest importance scores, calculated uniformly with each algorithm, were deemed as feature bands. As shown in Figure 5, under the conditions of no preprocessing and SG smoothing preprocessing, the selected feature bands by SPA, CARS, and random frog algorithms mostly fell in the front half of the wave region (420–580 nm), while the UVE algorithm tended to select information from the back half of the wave region. The fluorescence reaction in the front half of the wave region is mainly caused by the excitation of amylopectin, some vitamins in wheat flour, and DON substances. Therefore, it is believed that the useful information for wheat flour classification is mainly provided in the front half of the spectrum. After normalization, the random frog algorithm also selected more bands from the back half as feature bands, and the fluorescence information difference between the 600–800 nm bands of wheat flour samples with different DON contents was amplified, as shown by the normalized spectral curves.

### 3.4. RF and SVM Modeling Results

RF and SVM classification models were developed using fluorescence spectral data with diverse combinations of feature bands. Table 2 shows the classification results of each model. It can be observed that the performance of the models mainly improved following the implementation of feature band selection and preprocessing when compared to the full spectra modeling presented in Table 1. Nevertheless, certain models failed to perform well despite undergoing feature band selection without spectra preprocessing, indicating a decrease in recall and accuracy. The occurrence of such error may be attributed to the underlying noise in selected feature bands, significantly impacting model performance. Moreover, the false-positive rate of the SVM model was notably lower than that of the RF model. This demonstrates that the combination of feature band data in the SVM model is more effective in accurately identifying negative samples. In the models that combined preprocessing and feature selection processes, the SG–CARS–SVM, normalization–random frog–SVM, and SG–random frog–RF models all achieved classification accuracies ≥97% on the prediction set. The SG–CARS–SVM model performed the best, with a prediction set accuracy of 97.78% and a false-positive rate of 1.18%. The good performance of the model is linked to the preprocessing and feature band selection results. After SG smoothing, all the feature bands chosen by CARS fell in the region of 420–500 nm, which is in line with the detection results of DON fluorescence emission wavelengths near 460 nm. Upon normalization, the feature band selection of the random frog models shifted from the front half of the wave region to the back half, resulting in the improved performance of the SVM model. This may imply that DON can react with certain substances at this wavelength of fluorescence emission, leading to changes in fluorescence signal strength, and normalization preprocessing enhances this change. However, the SPA and UVE showed relative weakness in feature band selection in this study.

**Figure 6:**
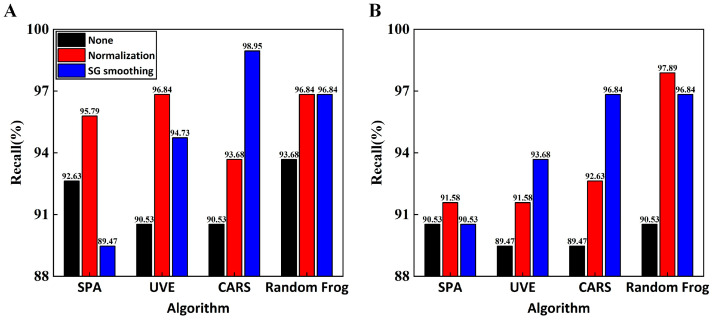
Comparison of recall rates of different models: (**A**) RF models; (**B**) SVM models.

In this study, the recall rate was a more accurate evaluation index of a model’s ability to detect positive samples when compared to the other two indicators. Figure 6 shows that the SG–CARS–RF model has the highest recall rate of 98.95%, which implies that this model is most adept in detecting positive samples and has a prediction set accuracy of 96.11%. Generally, the recall rates of RF models were higher than those of the SVM models, particularly for the models without normalization preprocessing. Additionally, the changes in the recall rate revealed that the performance of the RF model was more consistent across feature band combinations. This may be due to the fact that each decision tree in the RF algorithm is trained using a random sample with replacement of the original data, which improves the model’s robustness since it is more tolerant to outliers, primarily attributed to noise in the data.

**Figure 7:**
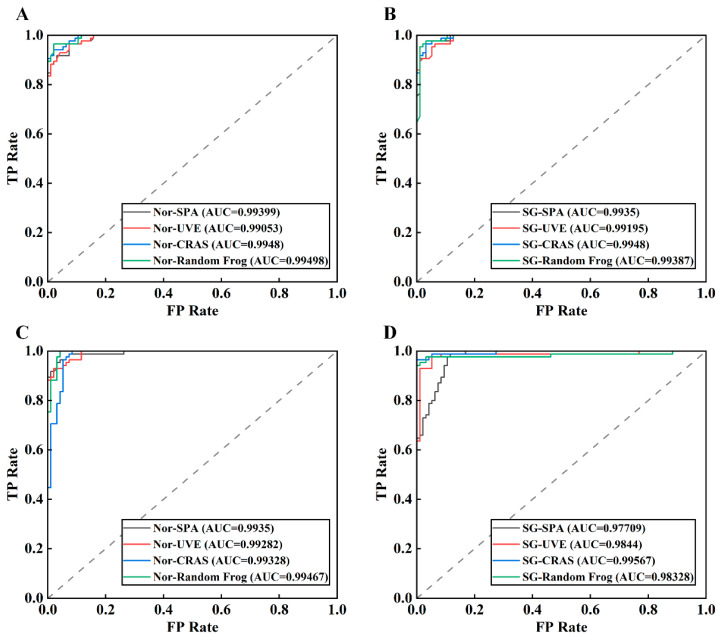
ROC curves of different models: RF models based on normalization (**A**) and SG smoothing (**B**) preprocessing combined with different feature selection methods; SVM models based on normalization (**C**) and SG smoothing (**D**) preprocessing combined with different feature selection methods.

As shown in Figure 7, ROC (receiver operating characteristic) curves were generated for the models developed using spectra with normalization and SG smoothing preprocessing. As the AUC (area under the curve) value approached 1, reflecting an ROC curve closer toward the upper left corner of the graph, the classification model performance was found to be better. Similar to the results from the recall rate indicator, the AUC value of the SVM model was observed to be relatively unstable. For instance, the AUC value of the SG–SPA–SVM model was 0.97709, while the AUC value of the SG–CARS–SVM model was 0.99567, relatively higher. The SG–SPA–RF and SG–CARS–RF models demonstrated a high level of robustness, as evidenced by their AUC values, which differed only slightly at 0.9935 and 0.9948, respectively. The shape of the curve indicates an imbalance between the FPR and TPR of the SVM models; in contrast, the ROC curves of the RF models concentrate more around the coordinate point (0,1) in the upper left corner, highlighting the high generalization ability of the RF classification model.

### 3.5. CNN Modeling Results

The CNN model was trained and tested using the same dataset. The model underwent 100 epochs of training using the adaptive moment estimation (Adam) optimization algorithm to adjust the model parameters with adaptive learning rates. The training started with an initial learning rate of 0.001. A regularization technique, L2 regularization, was used to reduce overfitting with a regularization rate of 0.0001. A segmented learning rate adjustment was used, which involved reducing the learning rate by half when the epoch number reached 75. To introduce variance in the training data, they were randomized during each epoch, considering that the training set was arranged in a sequence.

**Figure 8:**
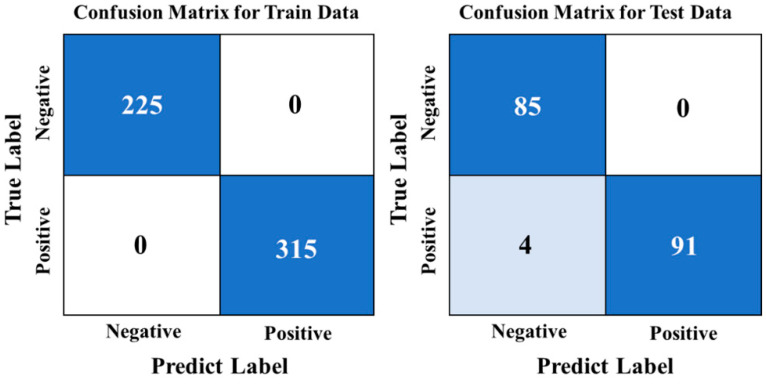
Confusion matrix of CNN model.

After testing the CNN model, its accuracy was found to be 97.78%, which is consistent with the SG–CARS–SVM model’s highest accuracy. The confusion matrix from the model testing is presented in Figure 8. It was discovered that all negative samples were correctly identified, leading to a false-positive rate of 0%. This accuracy is better than that of all previous models; however, four positive samples were misclassified as negative, resulting in a recall rate of 95.79%. This finding indicates that the performance of the CNN model may not be the best. This may be due to the strong learning ability of the CNN network, while the imbalance of sample size (the ratio of negative-to-positive sample size in the training set was 5:7) led to errors in CNN prediction. The CNN model’s training time was very short since the sample size was small, and the network structure was straightforward. Specifically, the model training reached high accuracy quickly, requiring only 13 s for 100 rounds of iterative training. This efficiency is much faster than the 50 s required for the SVM algorithm based on 20 bands.

### 3.6. t-SNE Visualization

**Figure 9:**
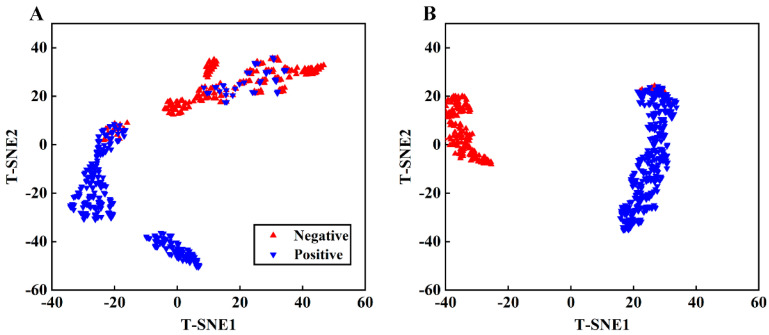
t-SNE visualization of original spectra (**A**) and extracted features from CNN (**B**).

As the convolutional layer transforms spectra from the original space into an abstract space, with a non-linear activation function, the interpretive quality of the CNN model is poor. This study used t-SNE for the two-dimensional visualization of both the original spectra and spectra after convolution, as shown in Figure 9. This figure displays that t-SNE effectively clustered the raw spectra for both samples. However, some positive samples and a few negative samples are crossed, corresponding to the peak intensity of the original spectra. Extracting data from the fully connected layer of the CNN network for t-SNE dimensionality reduction, as shown in Figure 9B, we found that the distances and clustering of both samples significantly improved, signifying that the CNN network extracted different features from the spectra for classification purposes.

## 4. Discussion

In this study, we developed and evaluated classification models using both commonly used machine learning methods (RF and SVM) and a deep learning method (CNN). When using machine learning methods, the raw spectra were first preprocessed, followed by feature band selection or spectral principal component extraction and classification with a classifier. This method has been widely used in machine learning and reported in the literature [47]. Conversely, the steps to create a deep learning model were relatively simpler. For example, convolutional layers in a CNN functionally include data preprocessing and feature extraction; the input is then fed to a predesigned network for classification. In both types of learning methods, dimensionality reduction is an important step. Nevertheless, they differ in terms of the approach they use to calculate the spectral features. While a convolution layer is used to calculate spectral features through sliding windows, a feature band selection algorithm selects feature wavebands as a subset of the original spectral bands, often linked to the fluorescence emission wavelength of the chemical substance. A convolutional layer can extract features and combine all spectral information from different bands, enabling it to better summarize the characteristics of the spectra. Both methods have their characteristics and advantages. In traditional processing, researchers can intuitively observe changes in fluorescence signals by analyzing raw and preprocessed spectra, and the selection of feature wavelengths indicates the corresponding spectral positions of changes in chemical composition, which is meaningful for experimental data analysis. Although both methods achieved high accuracy in this application, the SG–CARS–RF model exceeded in importance with its higher recall rate of 98.95%.

HSI technology is a highly promising and nondestructive high-throughput detection method. In comparison to diffuse reflectance hyperspectral imaging, FHSI is more sensitive to fluorescence signals, but it is still hampered by low signal-to-noise ratios. In this study, the intensity of the dark field noise reached one-fifth of the peak fluorescence intensity of the sample. Detecting weak fluorescent substances has always been challenging due to low signal-to-noise ratios. Researchers have attempted to mitigate the impact of low signal-to-noise ratios through various approaches. In terms of hardware, improving the quantum efficiency of spectral cameras and using more stable excitation light sources with higher intensity can boost the intensity of fluorescence emission, reduce integration time, and minimize system noise in the image. Nonetheless, it comes at a greater cost for detection. On the algorithmic front, developing preprocessing and dimensionality reduction algorithms that are tailored to specific needs can remove noise and enhance model accuracy. Deep learning models with numerous layers can attain remarkable precision. Nevertheless, overfitting is often a problem in this sort of research due to inadequate sample size. Overfitting reduces the ability of the model to generalize from the training data to unseen data, which will have a significant impact on the practical application of the model. For further research, data augmentation techniques like generative adversarial networks [48] can be explored to solve the problem of insufficient sample size.

In future applications, the equipment can be simplified based on the research results obtained in the lab, such as replacing the bulky xenon lamp with LED beads with fixed excitation wavelengths and swapping the hyperspectral camera with a miniaturized multispectral camera with only a dozen or even just a few spectral channels. In this way, the detection equipment can be portable. On the software side, the application of models is a challenge; practical applications involve many more interfering factors, such as environmental changes and variations in wheat flour quality, all of which require optimization through extensive experimental data in the laboratory. A highly targeted model can be eventually established and embedded into the detection equipment to achieve rapid and nondestructive detection.

Moreover, this study simulates natural infection in wheat by incorporating a minimal quantity of DON into wheat flour. The sample preparation process was subject to several unavoidable unstable factors resulting from experimental limitations, including the uneven mixing of wheat flour and DON solution; the formation of small lumps; the inconsistent spreading of the batter on the glass dish used for drying; and nonuniform drying, leading to variable moisture levels within the samples. These unpredictable factors might have some impact on fluorescence emission. Further improvement and optimization should be made in the follow-up research in this respect.

## 5. Conclusions

The present study utilized a combination of the FHSI technique and diverse modeling methods to effectively detect samples with excessive DON content. Twenty critical wavelengths were identified using the standardized and SG preprocessing algorithms, along with four feature band selection algorithms to establish SVM and RF classification models. The SG–CARS–RF and SG–CARS–RF models demonstrated better performance levels, with the highest recall rates of 98.95% and accuracy rates of 97.78%, respectively. By greatly minimizing noise and dimensionality, the SG preprocessing and CARS algorithms enhanced the performance of the model when compared to the original spectra-based model. Additionally, the CNN model exhibited a high accuracy rate of 97.78%. Employing t-SNE in visualizing the data in the original spectra and the fully connected layers of the CNN network, the findings proved that the convolutional network considerably enhanced the clustering relationship between the two types of samples. To summarize, the results of this study validate the feasibility of utilizing the FHSI technique and qualitative modeling methods in identifying samples with excessive DON concentration in wheat flour.

## Figures and Tables

**Table 1:** Model performance with different preprocessing algorithms.

Models	Preprocessing Algorithms	Test Accuracy	Recall	FPR
RF	None	93.33%	90.53%	3.53%
	Normalization	92.78%	90.53%	4.71%
	MSC	72.22%	91.58%	49.41%
	D1	86.67%	89.47%	16.47%
	SG	93.33%	89.47%	2.35%
	SG-1	90.00%	90.53%	10.59%
SVM	None	92.22%	91.58%	7.06%
	Normalization	95.56%	95.79%	4.71%
	MSC	82.78%	96.84%	31.76%
	D1	90.00%	91.58%	10.59%
	SG	92.78%	91.58%	5.88%
	SG-1	90.00%	90.53%	9.41%

**Table 2:** Model performance for different preprocessing and band selection algorithms.

Models	Preprocessing Algorithms	Band Selection Methods	Test Accuracy	Recall	FPR
RF	None	SPA	93.33%	92.63%	5.88%
		UVE	92.78%	90.53%	10.59%
		CARS	91.67%	90.53%	7.06%
		Random Frog	93.89%	93.68%	5.88%
	Normalization	SPA	93.89%	95.79%	4.71%
		UVE	93.33%	96.84%	4.71%
		CARS	93.33%	93.68%	4.71%
		Random Frog	93.89%	96.84%	2.35%
	SG	SPA	93.89%	89.47%	3.53%
		UVE	95.00%	94.73%	7.06%
		CARS	96.11%	98.95%	3.53%
		Random Frog	97.22%	96.84%	4.71%
SVM	None	SPA	94.44%	90.53%	3.53%
		UVE	90.50%	89.47%	3.53%
		CARS	95.00%	89.47%	4.71%
		Random Frog	93.89%	90.53%	2.35%
	Normalization	SPA	95.56%	91.58%	3.53%
		UVE	96.11%	91.58%	4.71%
		CARS	95.00%	92.63%	5.88%
		Random Frog	97.22%	97.89%	3.53%
	SG	SPA	92.78%	90.53%	2.35%
		UVE	93.89%	93.68%	3.53%
		CARS	97.78%	96.84%	1.18%
		Random Frog	96.11%	96.84%	2.35%

## Data Availability

The original contributions presented in the study are included in the article, further inquiries can be directed to the corresponding author.
